# Hypertrophy of the Pharyngeal Tonsil

**Published:** 1896-07

**Authors:** H. L. Hilgartner

**Affiliations:** Austin, Texas


For the Texas Medical Journal.

### HYPERTROPHY OF THE PHARYNGEAL TONSIL.

H. L. HILGARTNER, M. D., AUSTIN, TEXAS.

[Read before the Texas Medical Association, Fort Worth, Texas, April 29, 1896.]

THE subject I desire to broach for your consideration, "The Hypertrophy of the Pharyngeal Tonsil," while it is one of particular interest to the specialist, is also one of practical interest to the general practitioner. It seemed to me, therefore, preeminently fitted for discussion before this body. It is one of those diseases which comes so frequently under the notice of the physician in general practice as to make it peculiarly incumbent upon him to master its general theory, and to prepare himself to deal with its more usual symptoms and sequelæ. It has been the custom of specialists, when giving advice to the medical profession upon every topic, to indulge in a few glittering generalities about paliative treatment, but promptly to wind up with the caution to the general physician, after having acted upon these superfluous suggestions, to refer the patient to the specialist as the only hope of a permanent cure. I take the position that in this, as in other cases, the general practitioner is abundantly able to deal personally with the great mass of diseases of the kind referred to, and that if so, it is his duty, not only to himself, but to his patrons, to provide the treatment, which the specialist would arrogate to himself as his sole and individual prerogative. This is certainly a matter which the medical man in general practice is frequently called on to handle, and with regard to which it can not be maintained that any specialist training is prerequisite to an adequate and satisfactory treatment. The duty on the part of the general physician to meet this demand is all the more imperative when we consider the fact that the consequences of failure to provide timely treatment leads often to serious, and it may prove, irremediable consequences. It is a common experience of the medical man who comes in contact in the larger communities with a wider circle of patronage, to find especially in our public hospitals and asylums innumerable instances of the sad consequences which arise from allowing hypertrophy of the pharyngeal tonsil to go too long unattended to. We find these unfortunate consequences in their most pitiable shape in a total, or practically total, deafness. I therefore deem it appropriate to ask your indulgence while I undertake to outline the best results of modern theory and practice in this important and practical field.

To begin with, let us define the disease in question. It may be stated as follows: Hypertrophy of the pharyngeal tonsil is a true hypertrophy of the lymphoid structures found in the pharyngeal vault. The disease has been recognized since the days of William Hunter, and first fully described by Wilhelm Meyer, of Copenhagen, since whose exhaustive investigations little or nothing has been added to our knowledge of the disease or its treatment. It should be noted, however, that Czermiak both recognized and described the disease with considerable accuracy, but failed to discern its clinical seriousness.

*Etiology.*—It is well known to the profession that the disease is essentially incident to child life, being, perhaps, in the majority of cases, if not all, according to the best opinion, congenital. It tends to show itself on the wane as puberty advances, often disappearing after that time. As would naturally be expected, the lowered vitality which results from such causes as scrofula, syphilis or tuberculosis and the like, may predispose to this as to other similar affections. We may state that the immediate cause is to be found in the inflammatory changes superinduced most commonly by repeated colds upon the lining membrane of the air passages and their appendages.

The pathology of our subject I am compelled by limit of time to pass over rather hurriedly. The pharyngeal tonsil resembles very closely that of the fauces. It is composed of lymphatic and adenoid tissue, made up of stroma and lymph cells, the surface of which is covered with columnar, generally ciliated epithelium. The entire structure is quite vascular and like the faucial tonsil, has follicles.

*Symptomatology.*—The symptoms to which I propose to invite your attention, may be classified under six heads, the sixth of which is a general head, involving sundry symptomatic phenomena:

1. Excessive discharge of mucus and muco-pus.

2. The alteration in the vocal character, which may be simply defined as that incident to a cold in the head.

3. Ear complications, upon which, as I suggested in my opening remarks, special stress should be laid, for here, perhaps, the importance of remedial treatment is more urgent than elsewhere, since it involves, in many cases, the hearing of the patient. Chronic catarrhal otitis and chronic purulent otitis are the two aural conditions met with in adenoid disease. It is usually held that the ear complications are due to extension of the inflammatory conditions, or to pressure on the Eustachian orifice by masses of adenoid tissue.

4. Nasal stenosis with noisy respiration.

5. The peculiar facial appearance, consisting essentially in a broadening and flattening of the bridge and root of the nose. To this must be added the open mouth, caused by the nasal stenosis.

6. A general head under which I include various minor symptoms not elsewhere classified, viz: cough, headache, asthma, epistaxis, nightmare, etc.

*Diagnosis.*—There are four recognized methods of diagnosis: 1, the rhinoscopic mirror; 2, the nasal probe; 3, digital exploration; 4, the use of a spray of cosmoline or sweet oil, a method the value of which I would especially emphasize for the following reasons: Cosmoline or sweet oil, as you know, when sprayed into one nostril, the nasal passage and naso-pharynx being clear, will emerge from the opposite side in a stream. If, on the other hand, the naso-pharynx is obstructed, the spray either fails entirely to emerge from the other side, or escapes in a feeble stream.

*Prognosis.*—Prognosis, on the whole, is favorable.

*Treatment.*—As a general measure, of course, emphasis should be laid upon tonic treatment for the building up of the system, in which connection cod liver oil and general tonics are to be recommended. As to sprays, it will be found that little advantage results from their use, and that in no case will radical relief be secured thereby.

The preferable method of treatment is *complete extipiration of the growths*, a mode of procedure sure of permanent and satisfactory results in every case, when properly taken in hand. For this purpose three means are available—the unarmed finger, the forceps or the curette. Of these methods, either the use of the long-handled curette or the index finger will commend itself to the unpracticed operator, or the physician not expert in throat diseases.

Hemorrhage occurs rarely in a degree sufficient to demand interference. When it does occur, a tampon inserted into the pharyngeal vault is usually sufficient to control it.

Mr. President and gentlemen, I have aimed to present the salient points on the subject as clearly and succintly as the time allotted to such a paper would permit. Many interesting facts and ramifications of the topic have, of necessity, been passed over in silence, but I feel sure I can count upon your indulgence under the circumstances, if through hurry or inadventence I have omitted anything which you would have cared to have discussed in your hearing.

